# Assessment of the microbial load of airway clearance devices used by a cohort of children with cystic fibrosis

**DOI:** 10.1016/j.infpip.2021.100153

**Published:** 2021-06-06

**Authors:** B. Linnane, N.H. O'Connell, E. Obande, S.S. Dunne, C. Clancy, M.G. Kiernan, D. McGrath, K.J. O'Sullivan, L. O'Sullivan, C.P. Dunne

**Affiliations:** a Paediatric Cystic Fibrosis Department, University Hospital Limerick, Limerick, Ireland; b National Children's Research Centre, Crumlin, Dublin, Ireland; c Centre for Interventions in Infection, Inflammation & Immunity (4i) and School of Medicine, University of Limerick, Limerick, Ireland; d Department of Clinical Microbiology, University Limerick Hospital Group, Limerick, Ireland; e Rapid Innovation Unit – Confirm Centre for Smart Manufacturing, School of Design & Health Research Institute, University of Limerick, Limerick, Ireland

**Keywords:** Cystic fibrosis, Positive expiratory pressure (PEP), Oscillating intrapulmonary pressure (OPEP), Microbiology, Hygiene, Cleaning

## Abstract

**Background:**

Positive expiratory pressure (PEP) devices are an important element of the management of cystic fibrosis, and of other respiratory diseases. Whereas there have been reports in the literature of contamination of airway clearance devices and their surfaces by microbial pathogens, there is little evidence available regarding such contamination and its contribution to respiratory infection.

**Aim:**

To establish whether pathogenic bacteria can contaminate PEP devices in the context of normal cleaning and maintenance practices.

**Methods:**

Patients' home-use clearance devices were brought to a routine clinic appointment and collected for microbiology sampling and analysis. The patients were provided with replacement devices. Nineteen such devices were collected from 17 patients, reflecting use of multiple devices by some patients. Swabs were taken and cultured from each patient's used device, the patient's airway, as well as from new unopened and unused devices that acted as controls.

**Results:**

Seven of 19 devices (37%) tested positive for presence of pathogenic bacteria. Device-cleaning methods varied among patients and non-sterilization methods were found to be ineffective at removing pathogens. Microbial species found on the devices did not correlate with those identified from airway swabs.

**Conclusion:**

This study demonstrates the presence of pathogens on positive expiratory pressure devices. The potential for transmission of these pathogens to the patient's airway and the risk of infection remains unclear and requires further study.

## Introduction

Cystic fibrosis (CF) is a condition that affects the lungs, pancreas and intestines predominantly [1,2], leading to severe morbidity and early mortality [3]. It is most prevalent in Europe and North America, whereas East Asia has the lowest number of cases per capita [4]. Globally, Ireland has the highest prevalence of CF, with 2.98 cases per 10,000 population [[4], [5], [6]]. It has been predicted that the prevalence of CF in western European countries, including Ireland, could double by 2025, translating to a 22% increase in paediatric patients with CF and a 70% increase in adult patients with CF in Ireland [5].

CF is caused by the absence or malfunction of cystic fibrosis transmembrane conductance regulator (CTFR) protein, resulting from a mutation of the *CTFR* gene [3,7,8]. The CTFR protein regulates epithelial anion transport and mucociliary clearance [3,7], both of which are characteristically defective in CF [7]. The CF airway is, consequentially, a mucus-rich environment [9], optimal for bacterial colonization and growth [1,[10], [11], [12], [13]]. Therefore, respiratory infection with pathogenic bacteria, such as *Staphylococcus aureus*, *Haemophilus influenza*, *Burkholderia cepacia* complex, and *Pseudomonas aeruginosa*, occurs frequently in the CF airway [9,10,[12], [13], [14], [15]], resulting in recurrent chronic infection, decreased lung function, and accelerated respiratory disease [9,13,[16], [17], [18]].

The excess mucus present in the CF airway can cause air-flow restrictions, which can increase the work of breathing, create ventilation–perfusion mismatch, reduce gaseous exchange, and result in obstructions. Hence, airway clearance is an essential therapeutic procedure for patients with CF [[19], [20], [21]]. For the past 40 years, chest physiotherapy has been used to a great extent in clearance of obstructions and reduction in risk of infection or inflammation [20]. Conventional chest physiotherapy techniques include postural drainage with percussion and vibration (aided by a physiotherapist or relative), and forced expirations (huffing and coughing) [21]. However, these traditional methods of chest physiotherapy are time-consuming, physically demanding, uncomfortable, not aesthetically appealing, and are reliant on another person, all which can lead to a lack of adherence to the regimens [19,21].

Technological solutions have been explored to meet these challenges. Positive expiratory pressure (PEP) (e.g. TheraPEP®) and oscillating positive expiratory pressure (OPEP) (e.g. AerobikA®, Flutter®) devices (hereafter known collectively as PEP devices) have now become ubiquitous in the treatment of CF [[22], [23], [24]]. Such devices reduce mucus viscoelasticity, aid mucus mobilization, and subsequently promote mucus clearance, by forcing the airways open for longer during exhalation by increased resistance (PEP devices) or short pulses of resistance (oscillating intrapulmonary pressure) (OPEP devices) [19,23,[25], [26], [27], [28], [29], [30]]. When used correctly, these devices are proven to be as effective as traditional chest physiotherapy methods [21,[31], [32], [33]].

Inefficient cleaning and disinfection of PEP devices may pose a health risk to patients with CF, yet a consensus best practice standard for these regimens remains to be established. Cleaning is defined as the physical removal of foreign material, such as microbes, dirt and impurities from surfaces and objects, normally accomplished using water with detergents or enzymatic products, while the purpose of disinfection is to kill microbes on objects, usually achieved through chemical or thermal means [34]. Despite recommendations from the US Centers for Disease Control and Prevention (CDC) that PEP devices undergo ‘high-level disinfection’ [35], there are no official guidelines for cleaning or disinfecting PEP devices. Notably, the Cystic Fibrosis Foundation (CFF) has endorsed guidelines for cleaning and disinfection of aerosol therapeutic devices (nebulizers) [36,37]. However, manufacturers' recommendations for cleaning and disinfecting PEP devices can differ from those for nebulizers [22,[36], [37], [38]], and can vary greatly between devices [36]. Specifically, it has been noted that some PEP device manufacturers recommend to clean their device with tap water, which contradicts CFF guidelines for cleaning nebulizers [22,37,38]. Interestingly, cleaning with tap water has been proposed as a source of *Stenotrophomonas maltophilia* detected in a PEP device used by a CF patient [22].

To the best of our knowledge, only one previous published study has investigated the potential for bacteria to colonize PEP devices [38]. The authors of that study reported the presence of bacteria, including *Staphylococcus pasteuri*, *Staphylococcus warneri*, *Corynebacterium* spp., *Pseudomonas stuzeri*, *Moraxella* spp., and *Streptococci* spp., in 50% of devices tested after cleaning. That group did not observe an association between the bacteria found in the devices and those detected in the patients' sputum [38]. Given the high prevalence of CF in Ireland, and the now expansive range of pathogens detected in CF patient airways, the association between these core elements of CF therapy and the potential for harmful lung infection in this vulnerable population warrants further clarification. Accordingly, our study aimed to investigate the potential for PEP devices to act as reservoirs for bacteria in the context of normal cleaning, disinfection, and maintenance practices, and subsequently infect patients, in an Irish CF cohort.

## Methods

### Participants

This single-centre study was performed in an outpatient department (OPD) at a large regional tertiary hospital in Limerick, Ireland (UHL). Approval for this study was granted by the UHL Ethics Committee. Participants included a convenience sample of paediatric patients with CF, who were using a PEP or OPEP device, attending the OPD for routine clinic or annual review visits. There were no exclusion criteria for this study.

### Study design

Patients were asked to bring their current PEP device to a scheduled OPD appointment. To ensure that their normal routine of device maintenance and cleaning was not disrupted, patients were given no further instructions regarding the study prior to their OPD appointment. At the start of the clinic patients were introduced to the study, information was provided, and informed consent obtained. Information on the patient's normal device-cleaning practices was obtained from the parent or guardian, who was asked to complete a short survey (Supplementary Appendix) while at the clinic. Information on airway infections that occurred prior to and following their OPD appointment was obtained retrospectively. As per normal clinical practice, the patient's airway was swabbed and patient clinical status recorded during the appointment. In addition, the attending respiratory physiotherapist obtained cough swabs by asking the patient to cough on a cotton-tipped swab placed in, but not touching, the posterior pharynx.

With the patient's consent, their device was taken and a replacement device provided for future use. To reduce contamination during device handover and sampling, all handling of the patient's PEP device by researchers was performed using sterile gloves. The device mouthpiece was sealed with sterile latex. Sterile saline solution (0.9% w/v in H_2_O) was injected into the device until the device was filled. Following gentle agitation for 10 s, the seal was removed and the saline solution decanted into a sterile container. The saline solution was transferred to the microbiology laboratory immediately on ice for analysis.

### Bacterial identification

The wash saline solution was vortexed for 30 s and 100 μL (undiluted) was transferred on to tryptic soy agar (TSA) plates for culture and incubated for 48 h at 30°C ± 2. If colonies were present after incubation, one subculture was transferred and inoculated to each of the following media: Columbia blood agar, MacConkey, and TSA agar. These agar plates are used regularly to identify bacterial infection in patients with CF [39].

Further testing of the sample was used to confirm presence of indicated organisms (Enterobacterales, *P. aeruginosa*, or staphylococci) and enterococci. Samples underwent Gram staining. If bacteria were Gram positive, a catalase test was performed subsequently. Samples yielding a positive catalase test underwent Vitek 2 (bioMérieux, Marcy l’Etoile, France) biochemical testing for organism identification. Samples yielding negative catalase test were transferred to Slanetz and Bartley medium, on which a bile aesculin test was used to identify enterococci species. Gram-negative colonies were subjected to oxidase testing. Oxidase-positive colonies underwent Vitek biochemical identification. Oxidase-negative colonies that were lactose-fermenting were considered to be Enterobacterales. If the colonies were oxidase negative and a non-lactose fermenter, a Vitek biochemical identification was performed. A positive control was provided by culturing *S. aureus* NCTC 12981 on TSA agar. A negative control was established by filtering 100 mL of sterile water through a sterile funnel and placing the membrane filter on TSA agar.

Slides were prepared for Ziehl–Neelson staining (an acid-fast stain used to detect mycobacteria) [[40], [41], [42]]. In brief, carbolfuschin was applied to the wash saline solution on a slide, followed by heat fixation by passing the slide through a flame. The stained cell suspension was decolorized by applying acid alcohol for 20 s. Finally, a counterstain of Methylene Blue was applied to the slide for 30 s and the slide was rinsed and allowed to dry.

### Unopened device sampling and culture

Unused devices sealed in their original packaging were swabbed and used as a control. Unopened Aerobika® (Trudell Medical International, London, ON, Canada), TheraPep® (Smiths Medical, Minneapolis, MN, USA), and other devices were sampled for the presence of bacteria. The devices were removed from the packaging within a biosafety cabinet, without handling. All swabs (viscose) were submerged in 2 mL sterile phosphate-buffered saline (PBS) before use. The outside of the device and inside of the packaging were swabbed and the swabs were returned to the PBS. The exterior of the device was swabbed using sterile PBS, which was collected afterwards in a sterile dish. The inlet and outlet of the device were similarly swabbed and then flushed with sterile PBS, which was also collected in a sterile dish. The device was then fully disassembled, and the interior of the device swabbed and flushed with sterile PBS, as per other parts of the device. Note that this procedure differs from that described earlier for testing of patients' devices.

A sample of PBS (100 μL) gathered from the flush of each area of the device was transferred to agar and spread until dry. Swabs were vortexed for 5 s. Agar was also inoculated from the swabs using a simple streak. Additionally, PBS (100 μL) from the swab was transferred to the agar and, similarly, spread until dry. All samples were inoculated on to plate count agar (PCA) and Luria–Bertani (LB) agar, incubated at 35°C for 48 h, in aerobic and anaerobic conditions. Suitable control agar plates were also included to ensure that media (PCA and LB agar plates not opened in the biosafety cabinet), the environment (PCA and LB agar plates opened in the biosafety cabinet), and PBS (PCA and LB agar plates spread with sterile PBS that was used to flush the devices) used were sterile.

## Results

### Patient demographics

A total of 17 paediatric patients with CF were recruited, of whom six (35%) were female. The mean age of patients at the time of their OPD appointment was 9.8 ± 4.12 years (range: 3.8–17.5). The CF mutation F508del was found to be homozygous in 58% of patients, and heterozygous with a second disease causing mutation in the remainder.

### Survey results

Nineteen devices were used by the children, including Aerobika (*n* = 14), TheraPep (*n* = 4), and other (*n* = 1). Two patients used two devices concurrently: Aerobika and Thera-Pep; Aerobika and other devices. The devices were in use for a median of 6.0 ± 9.00 months (range: 3–48). Fourteen patients (82%) reported using their device(s) at least once a day (Table I).

**Table I:** Device cleaning and disinfection regimens and number of species cultured from each device

Device number	Patient number	Device type	Length of time with device (months)	Was the device used every day?	How many times a day is the device used?	How often do you clean your device?	What cleaning instructions were you given?	How do you clean your device?	Length of time to clean device (min)	How often do you sterilize your device?	How do you sterilize your device?	Length of time to sterilize your device (min)	No. of organisms cultured from device
1	1	Aerobika	12	Yes	1	Once a week	Wash with soapy water and air dry	Wash with soapy water and air dry	No info given	Once a week	Steam	–	7
2	2	TheraPep	12	Yes	2	Once a week	Wash with soapy water and air dry	Wash with soapy water and air dry	No info given	< once a month	Dishwasher	–	4
3	3	Aerobika	7	Yes	1	Never	Wash with soapy water and air dry	Boiling water in the microwave	No info given	Once a week	Sterilizing liquid	–	3
4	4	TheraPep	12	Yes	1	Never	Do not recall	Rinse with water and air dry	3	Never	N/A	N/A	4
5	5	TheraPep	4	Yes	1	After every use	Wash with soapy water and air dry	Wash with soapy water and air dry	2	< once a month	Microwave	5	5
6	6	Aerobika	12	No	N/A	After every use	Wash with soapy water and air dry	Wash with soapy water and air dry	1	After every use	Microwave	5	3
7	7	Aerobika	6	Yes	2	After every use	Wash with soapy water and air dry	Wash with soapy water and air dry	45	Never	N/A	N/A	5
8	8	Aerobika	3	Yes	2	Every second day	Do not recall	Wash with soapy water and air dry	2	Never	N/A	N/A	5
9	9	Aerobika	3	Yes	1	< once a month	Wash with soapy water and air dry	Wash with soapy water and air dry	20	< once a month	Microwave	5	2
10	9	TheraPep	48	Yes	1	< once a month	Wash with soapy water and air dry	Wash with soapy water and air dry	20	< once a month	Microwave	5	2
11	10	Aerobika	3	Yes	1	Never	Scrub with toothbrush and soapy water and air dry	Scrub with toothbrush and soapy water and air dry	2	Once a day	Boiling water	2	3
12	11	Aerobika	3	No	N/A	After every use	Wash with soapy water and air dry	Wash with soapy water and air dry	2	Never	N/A	N/A	3
13	11	Other	24	No	N/A	After every use	Wash with soapy water and air dry	Wash with soapy water and air dry	2	Never	N/A	N/A	3
14	12	Aerobika	12	Yes	1	After every use	Wash with soapy water and air dry	Boiling water	5	After every use	Boiling water	5	2
15	13	Aerobika	24	Yes	2	Never	Wash with soapy water and air dry	Wash with soapy water and air dry	15	Once a week	Sterilizing liquid	120	3
16	14	Aerobika	6	Yes	2	After every use	Wash with soapy water and air dry	Wash with soapy water and air dry	5	Once a day	Steam	5	4
17	15	Aerobika	6	Yes	2	–	Do not recall	Rinse under tap water	1	Once a week	Sterilizing liquid	20	4
18	16	Aerobika	3	Yes	2	< once a month	Boiling water and air dry	Boiling water and air dry	120	< once a month	Boiling water	120	4
19	17	Aerobika	3	No	N/A	After every use	Wash with soapy water and air dry	Wash with soapy water and air dry	9	After every use	Steam	14	4

–, no information; N/A, not applicable.

Twelve patients stated that they completed a cleaning regimen on their PEP device (14 devices), with eight devices (42%) cleaned after every use. Four devices (21%) were not cleaned. The remaining devices were cleaned every second day, weekly, or less frequently than once a month (Table I). Twelve devices (63%) were cleaned using the manufacturers' instructions for cleaning. The most common cleaning method used by the patients was to wash the device with soapy water and leave the device to air dry (86% devices). This cleaning regimen lasted an average of 10.8 ± 14.07 min. All but one of these devices were cleaned as per the manufacturers' instructions. One patient did not recall the manufacturer's cleaning instructions but rather cleaned the device with soapy water and allowed to air dry (device 8) (Table I). Overall, patients spent a median of 5.0 ± 18.00 min cleaning their device.

Two patients described using a presumed sterilization process instead of a regular cleaning regimen. Although both patients sterilized their devices using boiling water, one patient sterilized their device after each use (device 14) whereas the other sterilized their device less frequently than once a month (device 18) (Table I). Patients attempted to sterilize their device using a number of techniques: steam (*n* = 3, 21%), boiling water (*n* = 4, 29%), chemical (e.g. Milton) (*n* = 3, 21%) and microwaving (in hot water) (*n* = 4, 29%). The time that patients took to sterilize their device ranged from 2 to 120 min (median: 5.0 ± 15.00). Five devices were never sterilized by the patients or their caregivers. Of these, four were cleaned regularly (after every use or every second day) using soapy water. One device was neither cleaned nor sterilized (device 4) (Table I).

### Bacterial species present in patient devices

Bacteria were cultured from all patient devices. On average, four bacterial species were cultured from PEP devices (ranging from two to seven species). The majority of cultured bacteria were identified as normal respiratory tract flora; however, bacteria with pathogenic potential were cultured from seven devices (37%). The most common species cultured from devices were *Bacillus* spp. (*n* = 12), *Micrococcus* spp. (*n* = 9), and coagulase-negative staphylococci (*n* = 8) (Table II). Furthermore, *Stenotrophomonas maltophilia* was cultured from five devices, and *Curtobacterium flaccumfaciens*, *Kocuria rosea*, *Moroxella* spp. (not *catarrhalis*), *Paenibacillus glucanolyticus*, *Proteus* spp. and *Pseudomonas koreensis* were also cultured from ≤2 devices. There was no statistically significant difference between the mean number of bacterial species cultured from each device type (Aerobika 4.0 ± 1.33 vs TheraPep 4.0 ± 1.26 vs other 3.0; *P* = 0.867, one-way analysis of variance).

**Table II:** Bacteria cultured from used devices of patients with cystic fibrosis

Bacteria	Total (*n =* 19)	Devices			
		Aerobika (*n =* 14)	TheraPep (*n =* 4)	Other^a^ (*n =* 1)	*P*-value^b^
No. of devices from which bacteria were cultured	19 (100%)	14 (100%)	4 (100%)	1 (100%)	
*Bacillus* spp.	12 (63%)	10 (71%)	2 (50%)	NC^c^	0.298
Coagulase-negative staphylococci	8 (42%)	6 (43%)	1 (25%)	1 (100%)	0.395
*Curtobacterium flaccumfaciens*	1 (5%)	1 (7%)	NC	NC	0.828
*Kocuria rosea*	1 (5%)	1 (7%)	NC	NC	0.828
*Micrococcus* spp.	9 (47%)	2 (14%)	2 (50%)	1 (100%)	0.539
*Moroxella* spp. (not *catarrhalis*)	1 (5%)	1 (7%)	NC	NC	0.828
*Paenibacillus glucanolyticus*	2 (11%)	2 (7%)	NC	NC	0.671
*Proteus* spp.	1 (5%)	1 (14%)	NC	NC	0.828
*Pseudomonas koreensis*	1 (5%)	NC	1 (25%)	NC	0.138
*Stenotrophomonas maltophilia*	5 (26%)	4 (29%)	1 (25%)	NC	0.820

NC, not cultured.

a A device from a wide market selection, excluding Aerobika and TheraPep.

b χ^2^-Test comparing abundance of species cultured from various devices.

The length of time patients had owned their device, frequent usage, cleaning frequency, method, and length of time when cleaning had no discernible effect on whether bacteria were present in patient devices, the number of bacterial species in devices, or on the type of bacteria in devices (normal lung flora or pathogen). Similarly, sterilization frequency and length of time involved in the process had no effect on the same variables. The relatively low number of devices made it difficult to draw substantive conclusions from investigations of cleaning and sterilization methods, and their effect on bacterial colonization. However, some interesting observations that require further investigation were recorded. Notably, coagulase-negative staphylococci were not cultured from a device that was presumed sterilized using a microwave sterilization technique, contrasting with three of five devices that were found to be colonized having undergone a boiling-water-based sterilization technique.

### Microbial assessment of new unused devices

New unused Aerobika (*n* = 2), TheraPep (*n* = 2), and other (*n* = 1) devices were analysed to determine the presence of bacteria. *Staphylococcus epidermidis* was cultured from the PBS used to flush the inside of an Aerobika device, whereas *Bacillus mojavensis* (typically soil-borne) was detected on the inside of the packaging from a TheraPep device. There was no growth on control media (used to check the PCA and LB media, the environment, and the PBS used).

### Airway bacteria and device bacteria compared

Most patients had bacteria isolated from their airway (*n* = 15, 88%) at the time of sample collection at their OPD appointment. Normal lung flora were cultured from the majority of patient airways; however, pathogens were detected in the airways of five patients (29%). These included *S. aureus* (*n* = 3), *H. influenza* (*n* = 2), *P. aeruginosa* (*n* = 1), *Escherichia coli* (*n* = 1), and *Aspergillus fumigatus* (*n* = 1). Two patients had more than one pathogen cultured from their airways at the time of their OPD appointment. In each case, three bacterial species were co-isolated from the airways (*S. aureus*, *H. influenzae*, *E. coli* and *S. aureus*, *P. aeruginosa*, *A. fumigatus*). There were no common pathogenic bacteria found in both the patient airway, prior to and at the time of the OPD appointment, nor in the devices that they had used.

## Discussion

This single-centre small group study aimed to determine whether PEP devices may pose an airway infection risk for paediatric patients with CF by acting as pathogen reservoirs. There are previous reports indicating contamination of home-use airway devices with pathogenic species [[43], [44], [45]], and it has been demonstrated that species such as enterococci and staphylococci can survive for months on plastics [46] such as those used in the manufacture of PEP devices. Notably, PEP devices are not distributed typically as sterile devices, but rather have been manufactured in such a way as to eradicate or reduce their microbial load. However, links between device contamination and patient infection have not been clearly established in the literature and remain hypothetical. Proposed mechanisms mediating such infection include the transfer of environmental bacteria to the devices and subsequently into the patient airway during use, as well as the transfer of pathogenic species to the device from the patient airway and their reintroduction to the patient airway from the device, leading to recurrence following treatment (e.g. antimicrobial therapy for infection) [38]. For example, Greenwood *et al.* reported that 12 of their 60-participant cohort involving patients with CF and chronic *P. aeruginosa* infection were found to have pathogenic microbe contamination of nebulizer devices, while only one patient was found to have a correlating contamination of both their device and a sputum sample by the same bacteria species [44]. Similarly, our data did not find commonality between the bacteria collected from patient airway swabs and bacteria isolated from the patients' devices.

The presence of bacteria with pathogenic potential on seven of 19 devices tested as part of this study is a concerning finding. The multidrug-resistant, opportunistic bacterium *Stenotrophomonas maltophilia*, for example, which was found on five devices among our patient group, has been associated with hospital-acquired infections affecting patients with CF, among others [47]. Indeed, *S. maltophilia* is increasingly being isolated from the respiratory tract of patients with CF [48,49]. Interestingly, it was detected in an airway culture from a patient whose device was positive for *S. maltophilia* eleven months previously. Further, it has been determined as associated with infection caused by contaminated medical devices, including indwelling catheters and ventilation tubes [50], and previous studies have pointed to an association between reusable airway devices and transmission of *S. maltophilia* among CF patients [51]. Although no clear link between *S. maltophilia* and disease progression or mortality in CF has been established, these bacteria have been shown to cause pulmonary exacerbations, especially where a patient is immune-compromised or has a poor baseline lung function [50]. *S. maltophilia* has also been demonstrated to have a negative effect on recovery of CF patients following antibiotic treatment for pseudomonas infection [48].

Differences in PEP device cleaning and disinfection tech-niques may contribute to device contamination. Whereas a majority (63%) of patients in our study followed the manufacturers' cleaning instructions, sterilization was not common and some patients did not clean the device at all despite a majority of the participants using the device daily. Further, we noted that cleaning techniques that did not involve sterilization did not eradicate pathogenic bacteria effectively and that the different disinfection methods adopted may have had varying effects on separate pathogenic species. Given the prevalence of *Bacillus* spp. found, it is reasonable to lack confidence in these cleaning and disinfection practices affecting spore-formers, although we did not look specifically for spores. Overall, our findings correlate with data from another study in which cleaning achieved complete bacterial eradication in only 50% of devices [38]. It has been discussed elsewhere that manufacturers' advice regarding the frequency and technique required for device cleaning varies between manufacturers, and that the frequency at which PEP devices should undergo high-level disinfection is not clear from the CDC guidelines [38]. Given that context, it seems reasonable to suggest that healthcare staff may need to place heightened emphasis on clear cleaning and disinfection guidance at clinical appointments, providing unambiguous directions on technique and frequency. We recommend the discussion of this topic by Manor *et al.* who emphasize the importance of in-person demonstration of cleaning techniques [38]. It also appears prudent that manufacturers' guidelines ought to be updated, and subjected to regulation, if a clear link between device contamination and airway infection is established in future studies. Such clear and consistent guidance would likely improve patients' understanding and compliance.

### Limitations and recommendations

The limitation of this study is its small sample size, which limits generalizability to the CF patient population at large. We did not perform bronchoalveolar lavage, which could have provided a more accurate reflection of the presence of lower airway pathogens and which may or may not have determined a correlation with device microbial load. In addition, we did not sample devices or airways for viral or fungal pathogens. Further, we utilized conventional culture-based microbiology rather than more sensitive molecular techniques [45,52]. Despite this, we demonstrated presence of potential pathogens. A strength of this study is that patients were not informed that their devices would be assessed for microbial contamination. In a previous study, which attempted to establish the presence of contamination of PEP devices in a CF setting, the authors suspected that patients who were aware of their objective may have altered their cleaning regimen; consequently they observed a lesser microbial load than anticipated [38].

## Conclusions

This study demonstrates that PEP devices may act as reservoirs for potentially harmful microbial species, and that cleaning and disinfection techniques commonly used by patients do not ensure eradication of pathogenic bacteria from devices. Our data did not establish a clear link between bacteria found on PEP devices and those found in patients' airways, albeit that our sampling was limited in not including bronchoalveolar lavage. Thus, while there are studies showing transmission of pathogenic respiratory bacteria to both CF and non-CF patients from medical devices, there is no available evidence to definitively establish transmission specifically from PEP devices. More research is now needed, including larger group studies, to clarify whether PEP devices contribute to propagating infection in this susceptible patient population, both at home and during hospitalization, when they are vulnerable to nosocomial outbreaks and infection [52]. Further review of cleaning and disinfection practices and guidelines, notably those issued by device manufacturers, may be warranted in light of the varied and ineffective practices shown in this study. The evolving microbiology of CF-related respiratory infections, the increasing prevalence of CF, and the advantages of PEP devices over other respiratory therapies for CF make device-related pathogen transmission an important topic for future study.

## Credit author statement

B. Linnane: conceptualization, resources, writing – review and editing, supervision, funding acquisition. N.H. O'Connell: methodology. E. Obande: investigation. S.S. Dunne: methodology, writing – original draft, review and editing. C. Clancy: writing – original draft, review and editing. M.G. Kiernan: methodology, investigation, data curation, writing – original draft, review and editing. D. McGrath: methodology, resources, writing – review and editing, funding acquisition. K.J. O'Sullivan: conceptualization, investigation, resources, writing – review and editing, funding acquisition. L. O'Sullivan: conceptualization, resources, writing – review and editing, supervision, funding acquisition. C.P. Dunne: conceptualization, resources, data curation, writing – original draft, review and editing, supervision, funding acquisition.

## Conflicts of interest statement

B.L., D.McG., K.J.O.S., L.O.S. and C.P.D. are named inventors on patents resulting from the development of this research: EP 3 181 173 & US 10,806,876; and they were beneficiaries from the acquisition of the intellectual property from the University of Limerick, which occurred subsequent to the work described in this paper.

## Funding

This work was supported by an 10.13039/501100001588Enterprise Ireland research grant (CF-2016-0428-P) co-funded by the European Regional Development Fund 2014–2020.
